# First-degree atrioventricular block in patients with atrial fibrillation and atrial flutter: the prevalence of intra-atrial conduction delay

**DOI:** 10.1007/s10840-020-00838-3

**Published:** 2020-07-30

**Authors:** Florian Spies, Sven Knecht, Ivan Zeljkovic, Tobias Reichlin, Antonio Madaffari, Stefan Osswald, Christian Sticherling, Michael Kühne

**Affiliations:** 1 Department of Cardiology/Electrophysiology, University Hospital Basel, Basel, Switzerland; 2 Cardiovascular Research Institute Basel, University Hospital Basel, Petersgraben 4, 4031 Basel, Switzerland; 3 Department of Cardiovascular Diseases, Sestre milosrdnice University Hospital Centre, Zagreb, Croatia; 4 Department of Cardiology, Inselspital, Bern University Hospital, University of Bern, Bern, Switzerland

**Keywords:** First degree atrioventricular block, Intra-atrial conduction delay, Atrial fibrillation, Atrial flutter, HV interval

## Abstract

**Purpose:**

PR interval prolongation > 200 ms resulting in the diagnosis of first-degree atrioventricular block (AVB1) is caused by a delay in the AV nodal/His conduction and/or the right intra-atrial conduction (RIAC). The aim of the study was to assess the prevalence of AVB1 due to RIAC delay (AVB1 with normal AH and HV) in patients with atrial fibrillation (AF) and atrial flutter (AFlu).

**Methods:**

We included 1067 consecutive patients (33% female, age 63 ± 13 years) referred for catheter ablation of AF (AF-group) (453 patients), AF and AFlu (136 patients), AFlu (292 patients), and AVNRT/AVRT (186 patients). AH-, HV-, PR-interval, and *P*-wave duration were measured on the 12-lead ECG and the intracardiac electrograms in sinus rhythm. RIAC delay was defined as a prolonged PR interval > 200 ms with normal AH and HV intervals.

**Results:**

The prevalence of AVB1 is higher in patients with AFlu (41%) and AF (21%) and patients with both arrhythmias (30%) as compared with a reference group (8%) of patients with AVNRT/AVRT. AVB1 was due to RIAC delay in 42 of 67 patients (63%) in the AF-group, in 37 of 96 patients (39%) in the AFlu-group, and in 17 of 36 patients (47%) in the AF/AFlu group, respectively. AV nodal conduction delay was more common in AFlu patients compared with AF patients.

**Conclusion:**

RIAC delay is a common underlying cause of AVB1 in patients with AF and AFlu. These findings may impact the prescription of antiarrhythmic and AV-nodal blocking drugs.

**Electronic supplementary material:**

The online version of this article (10.1007/s10840-020-00838-3) contains supplementary material, which is available to authorized users.

## Purpose

First-degree atrioventricular block (AVB1), defined as PR interval longer than 200 ms, is frequently encountered in clinical practice and generally considered benign [1]. Since the PR interval is measured between the onset of the *P* wave and of the QRS-complex on the 12-lead ECG, it is dependent on both, the right intra-atrial conduction (RIAC) and the AV conduction over the AV node and the His bundle. The electrical and structural remodeling of the atrium observed in patients with AF [2, 4] and cavotricuspid isthmus (CTI)-dependent AFlu [3] may result in (right) atrial conduction delay influencing the PR interval measurements. The exact contribution of the RIAC interval on the PR interval commonly used for electrical characterization of the heart has not been systematically investigated in patients with AF and AFlu. Furthermore, the prevalence of RIAC delay defined as AVB1 with normal AH and HV intervals is unknown.

The aim of this study was to assess the prevalence of AVB1 due to RIAC delay by standard invasive electrophysiological measurements in patients referred for catheter ablation of AF and AFlu. In addition, patients undergoing catheter ablation of AVNRT or AVRT were analyzed as a reference group representing patients without any known or suspected right atrial myocardial conduction disease.

## Methods

We included 1067 consecutive patients undergoing an electrophysiological study for ablation of AF (AF-group), CTI-dependent AFlu (AFlu-group), and AVNRT/AVRT (reference-group). Diagnosis of CTI-dependent AFlu was performed by positive entrainment maneuver from the CTI in case of persistent flutter in the electrophysiology lab and based on 12-lead documentation showing a negative saw-tooth waves in inferior leads and positive waves in V1. Patients with documented AF in the AFlu-group and documented AFlu in the AF-group were combined in an AF/AFlu-group. Measurements on the 12-lead ECG and intracardiac electrograms using standard quadripolar and decapolar catheters with 5 mm and 2 mm interelectrode spacing (Abbot Medical and Biosense Webster, USA) were performed by two experienced technicians at the end of the procedure in sinus rhythm on the EP recording system (Sensis, Siemens, Germany). Intervals were defined as follows: atrium-His (AH) interval—onset of atrial signal to onset of His-deflection on the His recording (normal: 60–125 ms), His-ventricle (HV) interval—onset of His-deflection to onset of QRS interval (normal: 35–55 ms), *P*-wave duration—onset to offset of the *P* wave of all 12 leads, PR interval—beginning of *P* wave to first deflection of QRS, QRS duration—onset to offset of the QRS complex of all 12 leads, and RIAC—onset of *P* wave to onset of the atrial signal on His catheter. RIAC delay was defined as a prolonged PR interval > 200 ms with normal AH and HV intervals. The definition of persistent and paroxysmal AF was performed according to the AHA/ACC/HRS guidelines [5]. Patients treated with antiarrhythmic drugs (class IC and class III) were excluded.

## Results

A total of 858 patients (33% female, 63 ± 13 years) were analyzed. Thereof, 237 (28%), 319 (37%), 120 (14%), and 182 (21%) were included in the AFlu-group, the AF-group, the AF/AFlu-group, and the reference-group, respectively. Baseline data were significantly different between the groups (Table 1). Data of an age- and sex-matched analysis are shown in supplemental Table 1 and 2. The baseline data of the patients with paroxysmal and persistent AF were not different. AVB1 was present in 67 patients (21%) of the AF-group, in 96 patients (41%) of the AFlu-group, in 36 patients (30%) of the AF/AFlu-group, and in 15 patients (8%) of the reference-group. In the patients with AVB1, RIAC delay was observed in 42 of 67 patients (63%) in the AF-group, in 37 of 96 patients (39%) in the AFlu-group, and in 17 of 36 patients (47%) in AF/AFlu-group. The difference in RIAC delay between the AF-group (63%) and the AFlu-group (39%) was statistically significant (*p* = 0.013). PR interval prolongation was rare in the reference group, but if present was also due to RIAC delay in 40%. In patients without AVB1, the prevalence of a RIAC time > 45 ms [6] was 57%, 63%, 63%, and 27% in the AF-group, the AF/AFlu-group, the AFlu-group, and the reference-group, respectively.

**Table 1:** Baseline data and electrocardiographic data of the patients

All groups *n* = 858	AF-group *n* = 319 (37%)	AF/AFlu-group *n* = 120 (14%)	AFlu-group *n* = 237 (28%)	Reference-group *n* = 182 (21%)	*p* value
Baseline data					
Age (years)	61 ± 11 §, ‡, #	67 ± 11 ‡, †	68 ± 12 §,*	55 ± 19 #, *, †	< 0.001
BMI (kg/m^2^)	28 ± 5 #	28 ± 5 †	28 ± 5 *	26 ± 6 #, *,†	< 0.001
Female, *n* (%)	79 (25)	33 (28)	36 (15)	99 (54)	< 0.001
Paroxysmal AF, *n* (%)	195 (61)	51 (43)	n. a.	n. a.	n. a.
Hypertension, *n* (%)	194 (61)	67 (56)	95 (40)	59 (32)	< 0.001
Beta blockers, *n* (%)	239 (75)	86 (72)	109 (46)	47 (26)	< 0.001
Electrocardiographic data					
AH interval (ms)	84 ± 25 §	87 ± 28 ¥	100 ± 42 §, ¥,*	86 ± 26 *	< 0.001
HV interval (ms)	43 ± 8 §, #	45 ± 9 †	48 ± 11 §,	41 ± 7 #, *, †	< 0.001
*P*-wave duration (ms)	125 ± 19 §, #	131 ± 21 †	137 ± 27 §, *	108 ± 15 #, *, †	< 0.001
PR interval (ms)	179 ± 31 §, #	186 ± 37 ¥, †	202 ± 48 §, ¥, *	165 ± 30 #, *, †	< 0.001
RIAC interval (ms)	52 ± 17 #	54 ± 21, †	55 ± 22 *	38 ± 15 #,*, †	< 0.001
AH prolonged, *n* (%)	18 (6)	13 (11)	44 (19)	9 (5)	< 0.001
HV prolonged, *n* (%)	18 (6)	8 (7)	38 (16)	4 (2)	< 0.001
AVBI, *n* (%)	67 (21)	36 (30)	96 (41)	15 (8)	< 0.001
RIAC delay, *n* (%) (overall)	42 (13)	17 (14)	37 (16)	6 (3)	0.001
RIAC delay, *n* (%) (AVBI)	42 (63) §	17 (47)	37 (39) §	6 (40)	0.021

Values are shown as mean ± standard deviation. *P* value from ANOVA, with post-hoc Tukey test: § p < 0.05 between AF and AFlu; # *p* < 0.05 between AF and Reference-group; ‡ *p* < 0.05 between AF and AF/AFlu –group; *p < 0.05 between AFlu and reference-group; ¥*p* < 0.05 between AFlu and AF/AFlu -group; †*p* < 0.05 between AF/AFlu and reference-group

## Discussion

Whereas it is known that AV conduction is determined by the RIAC and the conduction over the AV node and the His bundle, its dispersion has never been quantified in such a large cohort of patients with AF and CTI-dependent AFlu. The main findings of this analysis are as follows:

1. The prevalence of AVB1 is high in patients with AFlu and AF and a combination of both arrhythmias as compared with a reference group of patients with AVNRT/AVRT.
2. The prevalence of RIAC delay as the cause of PR prolongation is significant in patients with AF, AFlu, and a combination of AF and AFlu, with the highest proportion observed in the AF group.

In previous studies, PR prolongation has been shown to be independently associated with the risk of developing AF [7] and all-cause mortality [8]. However, the individual contribution of the RIAC and the AV nodal conduction on the PR interval have not been investigated in this context. In consequence, whether the reported risk for these events is due to right atrial conduction delays or the delay within the AV node is unclear. To address this lack of knowledge, we analyzed patients with AF and AFlu to investigate whether differences in the site of the conduction impairment (RIAC or AV nodal) can be observed for these groups.

**Fig. 1:**
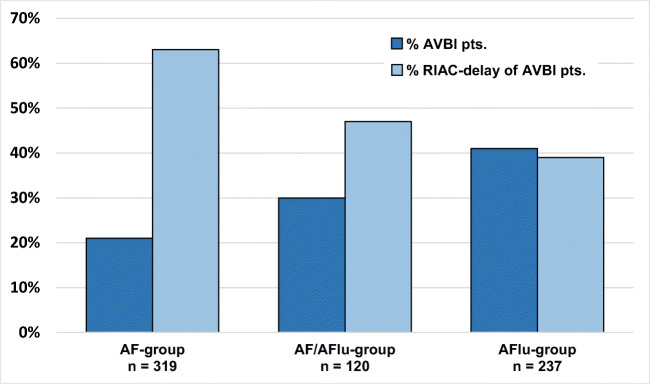
Bar chart of prevalence of AV-block first degree (AVBI) and right atrial conduction (RIAC) delay in patients with atrial fibrillation (AF), atrial flutter (AFlu), and the patients with both arrhythmias, AF and AFlu. The dark blue bar represents the percentage prevalence of AVBI in the overall cohort of the respective group; the light blue bar represents the percentage prevalence of RIAC delay in the cohort of AVBI patients of the respective group

In our patients, the prevalence of AVB1 defined by PR prolongation > 200 ms was relatively high in patients with AF (21%) and even higher in CTI-dependent AFlu (41%). Interestingly, RIAC delay as a measure of solely right intra-atrial conduction delay (without AV conduction delay) was observed more often in patients with AF than with AFlu, whereas the AFlu-group showed an AVB1 more often due to AV nodal conduction delay. This observation is in contrast to the only other study investigating electrophysiological difference in the right atrium between patients with AFlu and AF [9]. Medi et al. reported that patients with AFlu showed more advanced remodeling with slowed conduction and lower voltage areas especially in the posterior right atrium than with AF. However, this was a small study with only 10 and 13 patients in the AFlu and AF group, respectively, and the baseline details such as type of AF and prescription of AV blocking agents was not reported, which might explain the differences, as well as an inhomogeneous distribution of a right atrial remodeling (Fig. 1).

In addition, our findings may have additional clinical implications based on the differentiation between AV nodal-related versus RIAC-related conduction delay. For example, since the administration of negative dromotropic and/or antiarrhythmic drugs performed with caution in patients with AV nodal conduction disturbances [8], our findings show that the use of these drug therapies should not be limited because of AVB1 per se because the cause of AVB1 is a delay in the RIAC and not in the AV-node or His bundle in a significant number of cases. In conclusion, if the knowledge of the location of the atrioventricular conduction delay in patients with AVB1 is of clinical importance, an electrophysiological study might be indicated.

Based on our observations, one could hypothesize that AF results in more pronounced RA remodeling resulting in an increase of conduction delay compared with AFlu. This is in line with a basic research study in neonatal rat ventricular myocytes showing that irregular (AF-like) compared regular electrical activation (AFlu-like) results in a phenotype consistent with findings in atrial cardiomyocytes from AF patients [10]. Whether this knowledge about the differences in right atrial conduction delay should be used, e.g., in additional right atrial ablation strategies in patients with persistent AF or to perform AF ablation in patients with AFlu and RIAC delay due to the increased risk for AF [5] is an interesting research question that might be addressed in future studies. In this context, we await the results of the CRAFT study (https://clinicaltrials.gov/ct2/show/NCT03401099) investigating the impact of cryoballoon PVI in patients referred for catheter ablation of CTI-dependent flutter without previously documented AF.

## Limitations

The four groups differ with regard to baseline data. This is relevant especially for age, which was shown to correlate with the atrial conduction [11]. However, since the AFlu group was older than the AF group but the delay was more prevalent in the AF group, an older age in the AF group might not decrease this difference. The age- and sex-matched analyses shown in the supplemental tables confirm this observation. Furthermore, despite studying a large sample of 858 patients, the number of patients with RIAC delay in the different groups is relatively small. This was addressed by critical interpretation of the observations. One further limitation is the measurement of the PR interval itself and the absolute definition of a cut-off of 200 ms without addressing the heart rate dependency. Beta blockers were continued during the study; this can have an effect on the AV conduction. However, we focused our view on the RIAC delay, which by definition excluded patients with AH and/or HV extension. Also, despite not being observed or reported in other studies, an impact of the CTI ablation line on the PR interval cannot be excluded. Finally, our findings are observational in nature. Whether the underlying mechanisms can be explained mainly by remodeling associated with the respective arrhythmias, the predisposing risk factors, or whether specific molecular mechanisms such as connexin distribution or dysregulation are present [12], are currently unknown and requires further investigations.

## Conclusion

In conclusion, PR prolongation has a high prevalence in patients with AFlu and AF but frequently represents what we could call a “pseudo”-AVB1. In clinical practice, RIAC delay should be considered as a potential cause of AVB1 in any patient with PR prolongation and especially in patients with AF. The presented findings may help to discriminate the reason for AVB1 in patients with AFlu and AF and may have clinical implications when prescribing antiarrhythmic drugs, betablockers, or calcium-channel blockers or planning catheter ablation.

## Electronic supplementary material

ESM 1 (DOCX 16 kb)

ESM 2 (DOCX 16 kb)
