# Supplementary material for: First-degree atrioventricular block in patients with atrial fibrillation and atrial flutter: the prevalence of intra-atrial conduction delay
Source: J Interv Card Electrophysiol. 2020 Jul 30;61(2):421–5. doi: 10.1007/s10840-020-00838-3 (PMC8324594; doi:10.1007/s10840-020-00838-3)
Supplement: Supplementary file 1 — (DOCX 16 kb) [file 10840_2020_838_MOESM1_ESM.docx]

**Supplemental table 1 (age matched)**

| **All groups**  **n = 688** | **AF-group**  **n = 242**  **(35%)** | **AF/AFlu-group**  **n= 114**  **(17%)** | **AFlu-group**  **n= 209**  **(30%)** | **Reference-group**  **n= 123**  **(18%)** | **p-value** |
| --- | --- | --- | --- | --- | --- |
| **Baseline data** |  |  |  |  |  |
| Age [years] | 66 ± 7 | 66 ± 10 | 66 ± 11 | 66 ± 11 | 1.000 |
| BMI [kg/m^2^] | 28 ± 5 # | 28 ± 6 † | 28 ± 6 * | 27 ± 6 #, †, * | 0.065 |
| Female – n [%] | 70 (29) | 31 (27) | 29 (14) | 60 (49) | <0.001 |
| Paroxysmal AF – n [%] | 141 (58) | 50 (44) | n. a. | n. a. | n. a. |
| Hypertension – n [%] | 159 (66) | 63 (55) | 84 (40) | 53 (43) | <0.001 |
| Beta blockers – n [%] | 179 (74) | 82 (72) | 92 (44) | 42 (34) | <0.001 |
| **Electrocardiographic data** |  |  |  |  |  |
| AH interval [ms] | 85 ± 26 § | 88 ± 28 ¥ | 98 ± 40 §, ¥ | 90 ± 28 | <0.001 |
| HV interval [ms] | 43 ± 8 §, ‡ | 45 ± 9 ‡, ¥, † | 47 ± 11 §, ¥, * | 42 ± 7 †, * | <0.001 |
| P-wave duration [ms] | 128 ± 20 §, # | 131 ± 21 † | 135 ± 26 §, * | 112 ± 13 #, †, * | <0.001 |
| PR interval [ms] | 182 ± 33 §, # | 187 ± 38 ¥, † | 197 ± 45 §, ¥, * | 174 ± 31 #, †, * | <0.001 |
| RIAC interval [ms] | 54 ± 18 # | 54 ± 21 † | 53 ±21 * | 42 ± 15 #, †, * | <0.001 |
|  |  |  |  |  |  |
| AH prolonged – n [%] | 18 (7) | 12 (11) | 35 (17) | 8 (7) | <0.001 |
| HV prolonged – n [%] | 13 (5) | 6 (5) | 31 (15) | 4 (3) | <0.001 |
| AVBI – n [%] | 59 (24) | 35 (31) | 77 (37) | 14 (11) | <0.001 |
| RIAC delay – n [%] (overall) | 36 (15) | 17 (15) | 29 (14) | 6 (5) | 0.036 |
| RIAC delay – n [%] (AVBI) | 36 (61) § | 17 (49) | 29 (38) § | 6 (43) | 0.059 |

Values are shown as mean ± standard deviation. P-value from ANOVA, with post-hoc Tukey-test: §-p<0.05 between AF and AFlu; #-p<0.05 between AF and Reference-group; ‡-p<0.05 between AF and AF/AFlu –group; *-p<0.05 between AFlu and Reference-group; ¥-p<0.05 between AFlu and AF/AFlu -group; †-p<0.05 between AF/AFlu and reference-group.
