# Supplementary material for: First-degree atrioventricular block in patients with atrial fibrillation and atrial flutter: the prevalence of intra-atrial conduction delay
Source: J Interv Card Electrophysiol. 2020 Jul 30;61(2):421–5. doi: 10.1007/s10840-020-00838-3 (PMC8324594; doi:10.1007/s10840-020-00838-3)
Supplement: Supplementary file 2 — (DOCX 16 kb) [file 10840_2020_838_MOESM2_ESM.docx]

**Supplemental table 2 (gender matched)**

| **All groups**  **n = 696** | **AF-group**  **n = 309**  **(44%)** | **AF/AFlu-group**  **n= 112**  **(16%)** | **AFlu-group**  **n= 166**  **(24%)** | **Reference-group**  **n= 109**  **(16%)** | **p-value** |
| --- | --- | --- | --- | --- | --- |
| **Baseline data** |  |  |  |  |  |
| Age [years] | 61 ± 11 §, ‡, # | 66 ± 11 ‡, † | 69 ± 11 §, * | 56 ± 19 †, #, * | <0.001 |
| BMI [kg/m^2^] | 28 ± 5 # | 28 ± 6 | 28 ± 6 | 27 ± 6 # | 0.041 |
| Female – n [%] | 69 (22) | 25 (22) | 36 (22) | 26 (24) | 0.981 |
| Paroxysmal AF – n [%] | 186 (60) | 46 (41) | n. a. | n. a. | n. a. |
| Hypertension – n [%] | 188 (61) | 63 (56) | 71 (43) | 40 (37) | <0.001 |
| Beta blockers – n [%] | 231 (75) | 81 (72) | 71 (43) | 28 (26) | <0.001 |
| **Electrocardiographic data** |  |  |  |  |  |
| AH interval [ms] | 84 ± 25 § | 88 ± 28 ¥ | 100 ± 38 §, ¥, * | 85 ± 25 * | <0.001 |
| HV interval [ms] | 44 ± 8 § | 45 ± 9 ¥, † | 49 ± 11 §, ¥, * | 42 ± 7 †, * | <0.001 |
| P-wave duration [ms] | 126 ± 19 §, # | 131 ± 21 ¥, † | 140 ± 27 §, ¥, * | 111 ± 16 #, †, * | <0.001 |
| PR interval [ms] | 180 ± 31 §, # | 187 ± 38 ¥, † | 201 ± 46 §, ¥, * | 168 ± 31 #, †, * | <0.001 |
| RIAC interval [ms] | 52 ± 17 # | 51 ± 20 † | 54 ±22 * | 41 ± 14 #, †, * | <0.001 |
|  |  |  |  |  |  |
| AH prolonged – n [%] | 18 (6) | 10 (9) | 31 (19) | 5 (5) | <0.001 |
| HV prolonged – n [%] | 18 (6) | 8 (7) | 30 (18) | 2 (2) | <0.001 |
| AVBI – n [%] | 66 (21) | 33 (30) | 69 (42) | 11 (10) | <0.001 |
| RIAC delay – n [%] (overall) | 41 (13) | 17 (15) | 26 (16) | 5 (5) | 0.038 |
| RIAC delay – n [%] (AVBI) | 41 (62) § | 17 (52) | 26 (38) § | 5 (45) | 0.042 |

Values are shown as mean ± standard deviation. P-value from ANOVA, with post-hoc Tukey-test: §-p<0.05 between AF and AFlu; #-p<0.05 between AF and Reference-group; ‡-p<0.05 between AF and AF/AFlu –group; *-p<0.05 between AFlu and Reference-group; ¥-p<0.05 between AFlu and AF/AFlu -group; †-p<0.05 between AF/AFlu and reference-group.
